# Prevalence of Depression Among Patients With Type 2 Diabetes at King Abdullah Medical City

**DOI:** 10.7759/cureus.18447

**Published:** 2021-10-02

**Authors:** Wasayf Aljohani, Llojun Algohani, Anas Alzahrani, Mowaddah Bazuhair, Afnan Bawakid, Latifa Aljuid, Amin Al-ahdal

**Affiliations:** 1 Medicine, King Abdul-Aziz University, Jeddah, SAU; 2 Dentistry, Ibn Sina National College, Jeddah, SAU; 3 Medicine, Umm Al-Qura University, Makkah, SAU; 4 Biochemistry, King Abdul-Aziz University, Jeddah, SAU; 5 Family Medicine, King Abdullah Medical City, Makkah, SAU

**Keywords:** type 2 diabetes, depression, patient health questionnaire, neuropathy, hypertension, cardiovascular diseases

## Abstract

Introduction

Diabetes is one of the most common endocrine diseases worldwide. Type 2 diabetes and depression are commonly comorbid, high-prevalence, chronic disorders. Individuals with diabetes mellitus may have concurrent mental health disorders and are shown to have poorer disease outcomes. However, the evidence for clinical correlation is still unclear.

Objectives

To find the prevalence of depression among patients with type 2 diabetes at King Abdullah Medical City (KAMC).

Materials and methods

A cross-sectional observational study was conducted at KAMC in Makkah, the Kingdom of Saudi Arabia. Study participants were 267 people with type 2 diabetes (aged between 18-70 years). Those who had preexisting depression or drank alcohol, pregnant women, and postpartum women were excluded from the study. Participants’ clinical and demographic data and depression assessment were obtained on phone through an interview and using the Arabic version of the Patient Health Questionnaire-9 (PHQ-9), respectively. Glycated hemoglobin (HbA1c) test results of participants were collected electronically.

Results

The participants’ mean age was 57.88 ± 8.71 years, and 64.4% were males. Only 15.4% were current smokers and 16.9% were ex-smokers. The most common medical condition was hypertension (65.2%) and the majority (92.1%) had uncontrolled HbA1c with a mean value of 8.37 ± 1.92. Among the studied patients, 73% suffered from different degrees of depression; 36%, 19.9%, 8.6%, 5.2%, and 3.4% of the participants were suffering from minimal, mild, moderate, moderately severe, and severe depression, respectively. Only neuropathy was a significant risk factor of depression (odds ratio=2.87, 95% confidence interval=1.18-6.97, p=0.02).

Conclusion

Depressive symptoms are common in patients with uncontrolled type 2 diabetes who also suffer from neuropathy and retinopathy. Hypertension, cardiovascular diseases, and unhealthy diet had a significant correlation with depression.

## Introduction

Diabetes is one of the most widespread chronic diseases globally [1]. The International Diabetes Federation (IDF) estimates that 463 million people in the world have diabetes. Among these, 55 million live in the Middle East and North Africa region, and this figure is expected to reach 108 million by 2045 [1]. The Kingdom of Saudi Arabia (KSA) is one of the 21 participant countries and territories in the International Diabetes Federation Middle East and North Africa (IDF MENA) region. Approximately 18.3% of the total adult population in the KSA is diabetic [1]. The World Health Organization (WHO) states that depression is the second leading cause of loss of disability-adjusted life years [2]. Depression is a psychological disorder characterized by constant low mood, a sense of hopelessness, and several risk factors. The prevalence of depression in primary care varies between 15.3% and 22%, with a prevalence of up to 13% worldwide [3]. In KSA, the prevalence of depression ranges from 17% to 46%. Moreover, nearly 80% of primary care physicians fail to recognize 30-50% of depressed patients in their clinical practice [4].

Many studies in the KSA have shown that individuals with diabetes have a relatively high prevalence of depression. Depression was found to affect 37% of all diabetic patients in Northern Province’s Arar city [5]. Another study conducted in the Eastern Province’s Al Khobar city showed a prevalence of 48.7% [6]. In a 2018 study, Jazan city in the southern region reported a prevalence of 20.6% [7]. Another study showed a prevalence of 38.5% in the central region of Riyadh city [8]. In Jeddah and Makkah, a prevalence of 33.8% [9] and 21% [10], respectively, was reported.

There are several factors associated with depression in diabetic patients: comorbidities, diabetes-related complications, body mass index (BMI) ≥ 30 kg/m2, glycated hemoglobin (HbA1c) ≥ 8.0%, smoking, insulin treatment, and a long history of diabetes [11]. A cross-sectional study conducted at King Abdul-Aziz Medical City, Riyadh, KSA found old age, low income, nephropathy, hypertension, and heart failure as risk factors associated with depression among patients with type 2 diabetes [12].

Thus, there is a need to understand how depression and its associated factors affect people with type 2 diabetes [13]. More knowledge in this area will help to make better decisions to enhance healthcare provided to diabetic patients [13]. The present study focuses on estimating the prevalence of depression among patients with type 2 diabetes at King Abdullah Medical City (KAMC) in Makkah, KSA, and determine the associated factors.

## Materials and methods

A cross-sectional observational study was carried out at KAMC in Makkah, KSA from July 2020 to January 2021. Patients with type 2 diabetes (aged between 18 and 70 years) receiving follow-up treatment at outpatient clinics in KAMC participated in the study. Those who had preexisting depression or drank alcohol, pregnant women, and postpartum women were excluded from the study.

Sample size

Using the WHO sample size calculator, a sample size of 385 patients was considered adequate. The final sample comprised 267 patients who agreed to participate in the study.

Data collection

Data were collected using a predesigned questionnaire that included two sections. The first section included items on the demographic data of patients. Data in the second section included HbA1c results of patients were collected electronically and patient responses to the Arabic version of the Patient Health Questionnaire-9 (PHQ-9) [14] were obtained via phone. The Arabic version of PHQ-9 is a sensitive but not specific screening tool for depression in comparison to clinical diagnosis. The PHQ-9 is a self-administered scale used to measure depression. Items are rated from 0 to 3, according to the increased frequency of experiencing different mental health concerns, and the total score ranges from 0 to 27. The score is interpreted as indicating no depression or minimal, mild, moderate, moderately severe, or severe depression [14, 15].

Data analysis

SPSS version 26 (IBM Corp, Armonk, USA) was used to analyze the data. Qualitative data were expressed as numbers and percentages, and the Chi-square test (χ2) was used to analyze relationships between variables. Quantitative data were expressed as mean and standard deviation (mean ± SD), and the Mann-Whitney U test was performed for non-parametric variables. The Mann-Whitney U test was used after testing the normality of quantitative data. The normality was tested using the one-sample Kolmogorov-Smirnov test and the value of significance was less than 0.05. So, it was considered as not normally distributed data and the nonparametric Mann-Whitney U test was used. 

## Results

Table 1 details the demographic characteristics of participants. The mean age of patients was 57.88 ± 8.71 years, 64.4% were males, 85.85% were married, 90.6% had a Saudi nationality, 89.5% were Makkah residents, 39% had a university degree, and 35.2% had a monthly income < 5000 SAR (Saudi riyal). The majority of patients (94.4%) were living with family, 73% were homeowners and 41.9% were retired. Only 15.4% were current smokers and 16.9% were ex-smokers. 

**Table 1:** Patients’ demographic characteristics (N = 267)

N (%)	Variable
57.88 ± 8.71	Age
Gender	
172 (64.4)	Male
95 (35.6)	Female
Marital status	
229 (85.8)	Married
9 (3.4) 25 (9.4)	Divorced Widowed
4 (1.5)	Single
Nationality	
242 (90.6)	Saudi
25 (9.4)	Non-Saudi
Residency	
239 (89.5)	Within Makkah
28 (10.5)	Outside Makkah
Educational level	
104 (39)	University degree
72 (27)	Secondary and high school
47 (17.6)	Primary school
44 (16.5)	Illiterate
Monthly income	
94 (35.2)	< 5000 SR
78 (29.2)	5001–10000 SR
86 (32.2)	> 10000 SR
9 (3.4)	Not reported
Living status	
252 (94.4)	With family
15 (5.6)	Lives alone
Lives in	
195 (73)	Own house
68 (25.5)	Rented accommodation
4 (1.5)	Not Reported
Job status	
112 (41.9)	Retired
95 (35.6)	Unemployed
60 (22.5)	Employed
Smoking status	
181 (67.8)	Non-smoker
41 (15.4)	Current smoker
45 (16.9)	Ex-smoker

Table 2 presents the clinical characteristics of participants. Most patients took oral medications for diabetes (48.3%) and 28.5% took both insulin and oral medications. The most common medical conditions were hypertension (65.2%), cardiovascular disease (44.6%), retinopathy (29.6%), and endocrine disease (15%). The diet of more than half of the patients (52.1%) was a mix of healthy and unhealthy foods. Most of them (54.7%) did not engage in physical exercises and the majority (92.1%) had uncontrolled HbA1c with a mean value of 8.37 ± 1.92. The mean duration of diabetes, age at diagnosis, and BMI were 14.36 ± 8.8 years, 42.75 ± 12.19 years, and 32.88 ± 7.57 kg/m2, respectively. Most of the participants were obese (58.8%) and 92.1% had uncontrolled HbA1c.

**Table 2:** Distribution of patients according to clinical data, diet, and physical activity (N = 267) BMI = body mass index; HbA1c = glycated hemoglobin

N (%)	Variable
Medication type	
1 (0.4)	None
59 (22.1)	Insulin
129 (48.3)	Oral agents
76 (28.5)	Both
2 (0.7)	Lifestyle modification
Medical condition	
4 (15)	Endocrine disease
79 (29.6)	Retinopathy
174 (65.2)	Hypertension
65 (24.3)	Neuropathy
23 (8.6)	Asthma
119 (44.6)	Cardiovascular disease
33 (12.4)	Renal disease
9 (3.4)	Liver disease
78 (29.2)	Other
Diet	
65 (24.3)	Mostly healthy
139 (52.1)	Healthy and unhealthy
62 (23.2)	Mostly unhealthy
1 (0.4)	Not reported
Physical activity	
146 (54.7)	No exercise
28 (10.5)	Once per week
30 (11.2)	Twice per week
63 (23.6)	Three or more times per week
14.36 ± 8.8	Duration of diabetes (years)
42.75 ± 12.19	Age at diagnosis
32.88 ± 7.57	BMI
0 (0.0)	Underweight
35 (13.1)	Normal weight
75 (28.1)	Overweight
157 (58.8)	Obese
8.37 ± 1.92	HbA1c
21 (7.9)	Controlled
246 (92.1)	Uncontrolled

**Figure 1:**
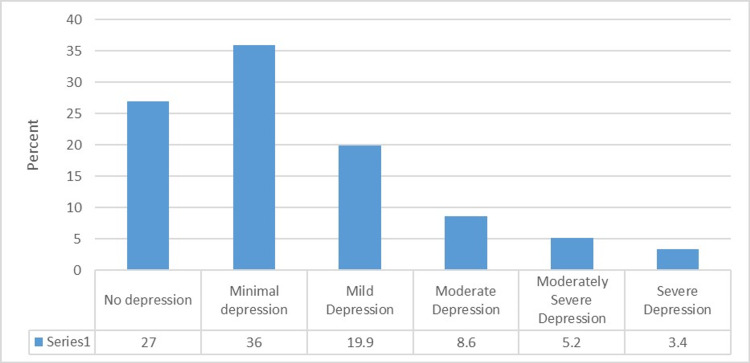
Percentage distribution of patients according to the prevalence of depression

Among the patients, 73% suffered from different degrees of depression. Figure 1 illustrates that 36%, 19.9%, 8.6%, 5.2%, and 3.4% of the patients suffered from minimal, mild, moderate, moderately severe, and severe depression, respectively. 

Table 3 demonstrates a non-significant relationship between depression among patients and their demographic characteristics (p ≥ 0.05).

**Table 3:** Table 3. Relationship between depression among patients and their demographic characters SES = socio-economic status

p-value	χ2	Depression N (%)	No depression N (%)	Variable
Gender				
0.859	0.03	125 (72.7)	47 (27.3)	Male
		70 (73.7)	25 (26.3)	Female
Marital status				
0.922	0.01	167 (72.9)	62 (27.1)	Married
		28 (73.7)	10 (26.3)	Not married
Nationality				
0.903	0.01	177 (73.1)	65 (26.9)	Saudi
		18 (72)	7 (28)	Non-Saudi
Residency				
0.84	0.04	175 (73.2)	64 (26.8)	Within Makkah
		20 (71.4)	8 (28.6)	Outside Makkah
Educational level				
0.58	0.3	74 (71.2)	30 (28.8)	Bachelor’s degree or higher
		121 (74.2)	42 (25.8)	Below bachelor’s
Income				
0.231	1.43	64 (68.1)	30 (31.9)	Low SES
		123 (75)	41 (25)	Not low SES
Living status				
0.567	0.32	185 (73.4)	67 (26.6)	With family
		10 (66.7)	5 (33.3)	Lives alone
Lives in				
0.352	1.3	138 (70.8)	57 (29.2)	Own house
		53 (77.9)	15 (22.2)	Rented accommodation
Job status				
0.351	0.86	154 (74.4)	53 (25.6)	Unemployed
		41 (68.3)	19 (31.7)	Employed
Smoking status				
0.3	2.4	129 (71.3)	52 (28.7)	Non-smoker
		34 (82.9)	7 (17.1)	Current smoker
		32 (71.1)	13 (28.9)	Ex-smoker

Table 4 shows that the prevalence of depression was higher in participants who suffered from retinopathy, neuropathy, and cardiovascular disease (p ≤ 0.05). 

**Table 4:** Relationship between depression among patients and their clinical data, diet, and physical activity (N = 267) BMI = body mass index; HbA1c = glycated hemoglobin; * = χ2

p-value	χ2	Depression N (%)	No depression N (%)	Variable
Medication type				
0.077	8.43	1 (100)	0 (0.0)	None
		51 (86.4)	8 (13.6)	Insulin
		88 (68.2)	41 (31.8)	Oral agents
		53 (69.7)	23 (30.3)	Both
		2 (100)	0 (0.0)	Lifestyle modification
Medical condition				
0.104	2.65	25 (62.5)	15 (37.5)	Endocrine disease
0.005	7.9	67 (84.8)	12 (15.2)	Retinopathy
0.547	0.36	125 (71.8)	49 (28.2)	Hypertension
0.001	11.44	58 (89.2)	7 (10.8)	Neuropathy
0.741	0.6	18 (77.3)	5 (22.7)	Asthma
0.025	5.03	95 (79.8)	24 (20.2)	Cardiovascular disease
0.117	4.29	29 (87.5)	4 (12.5)	Renal disease
0.279	1.18	8 (88.9)	1 (11.1)	Liver disease
Diet				
0.017	5.67	40 (61.5)	25 (38.5)	Healthy
		154 (76.6)	47 (23.4)	Unhealthy
Physical activity				
0.137	2.21	112 (76.7)	34 (23.3)	No exercise
		83 (68.6)	38 (31.4)	Does exercise
HbA1c				
0.86	0.03	15 (71.4)	6 (28.6)	Controlled
		180 (73.2)	66 (26.8)	Uncontrolled
0.596	0.53*	57.52 ± 9.2	58.87 ± 7.2	Age
0.601	0.52	4.46 ± 8.51	14.09 ± 9.56	Duration of diabetes
0.062	1.86	42.08 ± 12.32	44.54 ± 11.73	Age at diagnosis
0.338	0.95*	32.61 ± 7.58	33.65 ± 7.6	BMI
0.099	1.69*	8.48 ± 1.93	8.09 ± 1.86	HbA1c

Multivariate logistic regression analysis was performed to assess the independent predictors (risk factors) of depression among patients. Only neuropathy was found to be a risk factor (odds ratio = 2.87, 95% confidence interval = 1.18-6.97, p = 0.02) (Table 5).

**Table 5:** Multivariate logistic regression analysis of independent predictors (risk factors) of depression among patients HbA1c = glycated hemoglobin; CI = confidence interval

Odds ratio (95% CI)	p-value	Wald test	B	
1.82 (0.98–3.39)	0.057	3.62	0.6	Diet
1.55 (0.86–2.8)	0.142	2.16	0.44	Cardiovascular disease
1.54 (0.72–3.28)	0.26	.25	0.431	Retinopathy
2.87 (1.18–6.97)	0.02	5.45	1.05	Neuropathy
1.09 (0.4–0.263)	0.863	0.03	0.08	HbA1c control

## Discussion

Depression affects approximately one-third of people with diabetes [16], impacting their quality of life and presenting self-management challenges. Therefore, identifying mental health problems and associated factors among diabetic patients will assist healthcare physicians in providing better healthcare for such cases. A local study has suggested that patients with diabetes should undergo periodic psychological assessment as part of their clinical evaluation [13]. The current study estimates that 73% of patients with type 2 diabetes in Makkah, KSA, suffer from depression, which is a higher number than that reported by another study in Makkah (20.68%) in 2018 [16].

This variation in prevalence could be explained by the fact that the current study was conducted in a single facility at King Abdullah Medical City, which is a tertiary specialized hospital that deals with complicated cases and referred cases from other general hospitals. While the study conducted in 2018 included patients from three different institutions (Al Noor Hospital, the diabetes center of Hera'a hospital, and the Alaziziah outpatient clinic)

Additionally, differences in sample size could have played a role. The 2018 study involved patients suffering from two different types of diabetes, while this study focused only on type 2 diabetic patients, representing 90% of diabetics worldwide and in Saudi Arabia [16].

The prevalence of depression estimated in our study conforms with a study conducted in Tabuk city (78%) including 221 patients with type 2 diabetes [17]. Another study included 450 patients with type 2 diabetes attending primary healthcare centers, and the prevalence of depression was found to be 33.8% using the Depression, Anxiety and Stress Scale-21 (DASS-21) questionnaire [9]. The DASS-21 has a similar utility as the PHQ-9 in measuring the severity of depression symptoms [18]. Another study used the Hospital Anxiety and Depression Scale (HADS-D) and found 34.8% of 300 patients with type 2 diabetes in a hospital in Unaiza, Qassim region suffering from depression [19].

In another study conducted in the Eastern Province, KSA in 2013, depression prevalence was 49.6% [20].

The current study reported a higher prevalence of depression among diabetic patients than the studies conducted in Jazan (20.6%) [21] and Arar (37%) [5] that were both conducted in primary healthcare centers as well. A national-level meta-analytic study conducted in 2021 found the prevalence of depression was 38.06% in patients with type 2 diabetes (95% confidence interval = 30.84-45.28) [22].

Another point that could explain this variation in the result is the fact that this study was conducted during the COVID-19 pandemic, which had a huge psychological impact on the general population worldwide, leading to an increase in the global estimated prevalence of depression in general.

Furthermore, depression was associated with the prevalence of diabetes-related complications, which aligns with the findings ofother studies. Among these complications, only peripheral neuropathy was found to be significantly associated with depression. This finding confirms a study conducted at the primary healthcare centers in Qatif in 2011 [23].

Our findings demonstrated a significant association between depression and cardiovascular disease. Previous studies have also reported similar results [24]. According to our findings, physical activity lowers the likelihood of acquiring a psychiatric condition, such as depression, and type 2 diabetic patients who are physically inactive have a higher risk of depression, which is consistent with the findings of a study conducted in primary healthcare centers in Jazan in 2020 [25].

Patients with hypertension and ischemic heart disease were found to have a higher risk of developing depression than those with other comorbidities [25]. Increased diabetes duration is linked to a higher risk of complications and healthcare costs [26]. Neuropathy and retinopathy predicted a higher risk of depression than other diabetes-related complications during a study conducted in Arar [5].

The majority of our sample was male (64.4%), and our study concluded that there is no association between females and depression, unlike previous studies [27]. As 92.1% of our sample had uncontrolled HbA1c, we could not assess the association between HbA1c and depression. A link between poor glycemic control and depression has been discovered in several cross-sectional studies [28].

Limitations

This study has a few limitations. A cross-sectional design is insufficient for assessing the direction of the relationship between depression and diabetes in patients. Additionally, a single-center, hospital-based investigation with a randomly selected small sample size during a particular period of time does not provide reliable data about the prevalence of depression among patients with type 2 diabetes. Also, the fact that this study was conducted during the COVID-19 pandemic period could affect the results.

## Conclusions

Depression symptoms are common in patients with uncontrolled type 2 diabetes who also suffer from neuropathy and retinopathy. Hypertension, cardiovascular diseases, and unhealthy diet also had a significant correlation with depression. We advise that patients with uncontrolled type 2 diabetes who have these diabetes-related complications be regularly screened for depression.

Furthermore, most patients with uncontrolled type 2 diabetes were found to suffer from neuropathy; therefore, we recommend proper screening for depression among diabetic patients with neuropathy and early screening for neuropathy in type 2 diabetic patients to prevent depression because of late management of these complications.
